# Aggravation of functional mitral regurgitation on left ventricle stiffness in type 2 diabetes mellitus patients evaluated by CMR tissue tracking

**DOI:** 10.1186/s12933-021-01354-y

**Published:** 2021-07-31

**Authors:** Yi Zhang, Wei-feng Yan, Li Jiang, Meng-ting Shen, Yuan Li, Shan Huang, Ke shi, Zhi-gang Yang

**Affiliations:** Department of Radiology, West China Hospital, Sichuan University, 37# Guo Xue Xiang, Chengdu, 610041 Sichuan China

**Keywords:** Type 2 diabetes mellitus, Functional mitral regurgitation, Left ventricle, Strain, Magnetic resonance imaging

## Abstract

**Background:**

Functional mitral regurgitation (FMR) is one of the most common heart valve diseases in diabetes and may increase left ventricular (LV) preload and aggravate myocardial stiffness. This study aimed to investigate the aggravation of FMR on the deterioration of LV strain in type 2 diabetes mellitus (T2DM) patients and explore the independent indicators of LV peak strain (PS).

**Materials and methods:**

In total, 157 T2DM patients (59 patients with and 98 without FMR) and 52 age- and sex-matched healthy control volunteers were included and underwent cardiac magnetic resonance examination. T2DM with FMR patients were divided into T2DM patients with mild (n = 21), moderate (n = 19) and severe (n = 19) regurgitation. LV function and global strain parameters were compared among groups. Multivariate analysis was used to identify the independent indicators of LV PS.

**Results:**

The T2DM with FMR had lower LV strain parameters in radial, circumferential and longitudinal direction than both the normal and the T2DM without FMR (all *P* < 0.05). The mild had mainly decreased peak diastolic strain rate (PDSR) compared to the normal. The moderate had decreased peak systolic strain rate (PSSR) compared to the normal and PDSR compared to the mild and the normal. The severe FMR group had decreased PDSR and PSSR compared to the mild and the normal (all *P* < 0.05). Multiple linear regression showed that the regurgitation degree was independent associated with radial (β = − 0.272), circumferential (β = − 0.412) and longitudinal (β = − 0.347) PS; the months with diabetes was independently associated with radial (β = − 0.299) and longitudinal (β = − 0.347) PS in T2DM with FMR.

**Conclusion:**

FMR may aggravate the deterioration of LV stiffness in T2DM patients, resulting in decline of LV strain and function. The regurgitation degree and months with diabetes were independently correlated with LV global PS in T2DM with FMR.

**Supplementary Information:**

The online version contains supplementary material available at 10.1186/s12933-021-01354-y.

## Background

According to an epidemiological report in 2018, ~ 451 million people worldwide suffered from diabetes and 374 million suffered from impaired glucose tolerance in 2017, which is expected to increase to 693 million in 2045 [1, 2]. Functional mitral regurgitation (FMR) is the most common heart valve disease in diabetes and may increase the mortality of this population. A Verona diabetes study has shown that ~ 32% of type 2 diabetes mellitus (T2DM) patients have FMR. Compared with T2DM without FMR, T2DM with mild FMR have a 3.3-fold increase in all-cause mortality, while T2DM with moderate to severe FMR have a 5.1-fold increase in all-cause mortality [3]. Diabetic cardiomyopathy may cause LV remodelling, leading to LV enlargement, left ventricular enlargement, mitral annular dilation, papillary muscle displacement, and mitral valve insufficiency, thus resulting in FMR [4]. T2DM combined with FMR may increase left ventricle (LV) preload and force the ventricle to work under higher pressure, which may aggravate LV myocardial stiffness. Most of T2DM patients with FMR are asymptomatic and often neglected. Therefore, it is of great significance to investigate the cardiac dysfunction in T2DM patients with FMR before the occurrence of adverse events to reduce cardiovascular risk and improve outcomes.

Cardiac magnetic resonance (CMR) tissue-tracking technology based on the CMR cine sequence can provide a “one-stop-shop” evaluation of LV systolic or diastolic dysfunction [5]. CMR derived strain has been proved to be able to detect subclinical myocardial deformation prior to the decrease of left ventricular ejection fraction (LVEF). It can provide early reference for the diagnosis, detection and treatment of myocardial injury to improve the prognosis of high cardiovascular risk patients [6–8]. At the same time, the role of strain in predicting cardiovascular events has also been confirmed [9, 10]. However, to the best of our knowledge, few studies have used this technique to evaluate the additive effect of FMR on myocardial stiffness in T2DM patients. Therefore, this study aimed to investigate the characteristics of LV volume, function and strain in T2DM patients with or without FMR, to explore whether FMR aggravates the deterioration of LV stiffness in T2DM patients, and to identify the independent factors that affect the global peak strain (PS) of LV.

## Materials and methods

### Study population

From September 2016 to September 2020, 207 patients who were clinically diagnosed with T2DM (based on the current American Diabetes Association guidelines) and underwent CMR examinations in our hospital were included in the present study [11]. Exclusion criteria included ischaemic heart disease (n = 16), rheumatic heart disease (n = 4), congenital heart disease (n = 2), other primary cardiomyopathy (n = 9), other heart valve diseases (n = 10), contraindications to magnetic resonance imaging (allergy to contrast medium, metal implants or claustrophobia, n = 3) and poor image quality caused by arrhythmia (n = 6). Finally, a total of 157 T2DM patients with an average age of 58.3 ± 11.7 years and BMI of 24.6 ± 3.7 kg/m^2^ were included. Among them, 98 cases (62.4%) were free from FMR, and 59 cases (37.6%) had FMR (21 mild cases, 19 moderate cases and 19 severe cases). In addition, 52 age- and sex-matched normal individuals were recruited in the control group (36 males and 16 females; mean age, 55.5 ± 6.8 years; and BMI, 23.1 ± 3.6 kg/m^2^). The inclusion criteria of the control group were as follows: no history of impaired glucose tolerance, ischaemic heart disease, known systemic diseases (such as hypertension and hyperlipidaemia), abnormal ECG, abnormal ventricular motion and decreased LVEF detected by CMR.

Data on demographic characteristics, including sex, age, height, weight, blood pressure, resting heart rate, fasting plasma glucose, glycated haemoglobin (HbA1c), months with diabetes (months), total cholesterol, triglycerides, high-density lipoprotein (HDL), low-density lipoprotein (LDL) and antidiabetic drugs (biguanides, sulfonylureas, α-glucosidase inhibitors, glucagon-like peptide-1/dipeptidyl peptidase-4 inhibitors, sodium-glucose cotransporter 2 inhibitors and insulin), were collected from the digital medical records of the patients. Baseline information of normal volunteers was collected before the CMR scans. Body mass index (BMI) was calculated as weight (kg) divided by the square of height (m) [12]. Blood pressure and resting heart rate were recorded as an average of three measurements in the right arm in a sitting position after a 10-min resting period.

### CMR protocol

CMR examination was conducted using a 3.0T whole-body scanner (Skyra; Siemens Medical Solutions, Erlangen, Germany) with a 32-channel body phased-array coil in the supine position. Data acquisition was performed during the breath-holding period at the end of inspiration. A series of 8–12 continuous short-axis views of LV from the mitral valve to the level of the LV apex, were obtained using steady-state free precession with the following parameters: repetition time, 33.22 ms; echo time, slice thickness, 8.0 mm; flip angle, 39°; field of view, 234 × 280 mm^2^; 1.31 ms; and matrix size, 208 × 139 pixels.

### Image analysis

#### Calculation of LV volume and function parameters

Image analysis was performed by two experienced radiologists with more than 3 years of CMR experience using commercial software (cvi42; Circle Cardiovascular Imaging, Inc., Calgary, AB, Canada). The endo- and epicardial contours of the LV and right ventricle (RV) were delineated manually per slice on the end-diastole and end-systole images, and the papillary muscles and moderator bands were carefully excluded (Additional file 1). The LV volume and function parameters, including end-diastolic volume (EDV), end-systolic volume (ESV), LV stroke volume (LVSV), LV ejection fraction (LVEF), LV mass and RV stroke volume (RVSV), were automatically computed.

#### Identification and classification of FMR in T2DM patients

Mitral regurgitation could be identified on on the short axis, four chamber long axis and two chamber long axis views as the limited closure of the mitral valve orifice and a high-speed black retrograde signal from the LV to the left atrium through the mitral valve during ventricular systole [13]. The regurgitation fraction (RF) was calculated by using LVSV and RVSV according to the following formula: regurgitation fraction (RF) = (LVSV − RVSV)/LVSV (Additional file 2). According to the calculated results, DM patients with FMR were further divided into mild (RF < 30%), moderate (30 ≤ RF < 50%) and severe (RF ≥ 50%) regurgitation [13, 14].

#### LV strain analysis

Every voxel of myocardium was tracked on the short-axis, horizontal 4-chamber long-axis and vertical 2-chamber long-axis cine slices. The software automatically analysed the global LV strain variables, including peak strain (PS), peak systolic strain rate (PSSR) and peak diastolic strain rate (PDSR). Each strain parameter had three values in different directions. Radial strain was considered positive due to the thickening of the myocardium when the LV was contracting. Circumferential and longitudinal strains were considered negative because the myocardium shortened when contracting [15].

#### Reproducibility of LV strain

The intra-observer variability in the LV global strain parameters was assessed by an experienced investigator by comparing the measurements from 60 randomly selected cases analysed by the same observer after one month. The inter-observer variability was evaluated by comparing the measurements from the same population by another independent double-blinded experienced observer.

### Statistical analyses

Differences in Fasting plasma glucose, HbA1c, months with diabetes, blood lipids were analysed using Mann–Whitney *U* test and the difference in antidiabetic drugs were analysed using Chi square test between T2DM patients with and without FMR as appropriate. Continuous variables with normal distribution are presented as the mean ± standard deviation and median (25–75% interquartile range) for those with continuous non-normal distribution. Comparisons between three groups were made with one-way analysis of variance (ANOVA). The frequency (percentage) was used to represent the categorical variables, and the Chi-square test was used to compare the constituent ratio between different groups. Spearman’s test was used to analyse the correlation between LV global PS (radial, circumferential and longitudinal), regurgitation degrees, months with diabetes and blood lipids. The absolute values of circumferential and longitudinal strains were used in the correlation analysis to eliminate the confusion caused by the negative sign of circumferential and longitudinal strains. Variables with P < 0.1 and no collinearity in univariate analysis were included in the stepwise multiple linear regression model adjusted for sex, age, BMI, systolic blood pressure and resting heart rate. Inter-observer and intra-observer variabilities were assessed by the intraclass correlation coefficient (ICC). All analyses were performed by a two-tailed test, and P < 0.05 was considered statistically significant.

## Results

### Baseline characteristics

The baseline characteristics are shown in Table 1. The average age of T2DM patients with FMR was 60.7 ± 11.9 years old, which was significantly older than that of the normal control group (55.5 ± 6.8 years old) (P < 0.05). All T2DM patients had significantly higher weight and BMI than normal subjects (all P < 0.05). T2DM patients with FMR were older [60.7 ± 11.9 years vs. 57.0 (52.0–64.0) years] and had a longer months of diabetes [12.5 ± 5.4 months vs. 7.0 (4.0–11.0) months] than T2DM patients without FMR (all P < 0.05).

**Table 1:** Baseline characteristics of the study cohort

	Normal (n = 52)	T2DM	
		Without FMR (n = 98)	With FMR (n = 59)
Sex, male (%)	36 (69.2)	68 (69.4)	36 (61.0)
Age, years	55.5 ± 6.8	57.0 (52.0, 64.0)	60.7 ± 11.9*†
Height, cm	160.9 ± 6.7	166.5 (158.0, 170.3)*	164.0 (158.0, 169.0)
Weight, kg	57.5 (53.0, 63.0)	65.8 ± 10.5*	66.1 ± 9.8*
BMI, kg/m^2^	22.5 (20.5, 24.5)	24.2 ± 2.8*	24.7 (23.0, 26.7)*
SBP, mmHg	119.2 ± 5.3	126.5 (116.0, 135.3)*	120.0 (110.0, 138.0)
DBP, mmHg	80.0 (75.0, 83.0)	79.8 ± 12.5	77.7 ± 18.3
Rest heart rate, bmp	74.2 ± 9.9	77.5 ± 112.5	74.8 (67.4, 83.4)
Fasting plasma glucose, mmol/l	–	8.8 (6.8, 11.1)	8.0 (7.0, 10.0)
HbA1c, %	–	7.6 (6.7, 8.5)	8.2 ± 1.5
Months with diabetes, months	–	7.0 (4.0, 11.0)	12.5 ± 5.4†
Blood lipids			
TC, mmol/l	–	4.30 (3.5, 5.1)	4.1 (3.2, 4.7)
TG, mmol/l	–	1.4 (1.0, 2.1)	1.6 (1.1, 2.3)
HDL, mmol/l	–	1.1 (0.9, 1.4)	1.1 ± 0.4
LDL, mmol/l	–	2.3 (1.8, 3.0)	2.3 (1.5, 2.8)
T2DM with mild regurgitation, n (%)	–	–	21 (35.6)
T2DM with moderate regurgitation, n (%)	–	–	19 (32.2)
T2DM with severe regurgitation, n (%)	–	–	19 (32.2)
Antidiabetic drugs, n (%)			
Biguanides	–	51 (52.0)	29 (49.2)
Sulfonylureas	–	6 (6.1)	9 (15.3)
α-Glucosidase inhibitor	–	23 (23.5)	15 (25.4)
GLP-1/DPP-4 inhibitor	–	9 (9.2)	9 (15.3)
SGLT2 inhibitor	–	12 (12.2)	5 (8.5)
Insulin	–	35 (25.7)	22 (37.3)
eGFR, ml/min/1.732 m^2^	–	79.2 ± 30.4	73.7 ± 26.2

*T2DM* type 2 diabetes diabetes mellitus, *FMR* functional mitral regurgitation, *BMI* body mass index, *SBP* systolic blood pressure, *DBP* diastolic blood pressure, *HbA1c* glycated haemoglobin, *TC* total cholesterol, *TG* triglycerides, *HDL* high-density lipoprotein, *LDL* low-density lipoprotein, *eGFR* estimated glomerular filtration rate

*P < 0.05, T2DM vs. Normal; †*P* < 0.05, T2DM with FMR vs. T2DM without FMR

### Characteristics of LV volume, function and strain in T2DM patients with or without FMR

The patients without FMR had significantly higher LV mass and lower PS (radial and longitudinal), PSSR (radial), and PDSR (radial, circumferential) than that of normal subjects, while the LVEDV, LVESV, LVSV, and LVEF were similar to that of normal subjects (all P < 0.05).

In T2DM patients with FMR, the LVEDV, LVESV and LV mass were all higher than those in T2DM patients without FMR and normal subjects (all P < 0.05). The LVEF of T2DM patients with FMR was significantly lower than that of T2DM patients without FMR and the normal control group (all P < 0.05). By comparing strain variables, the LV global PS, PSSR, and PDSR in three directions were all significantly decreased in T2DM patients with FMR compared to both normal controls and T2DM without FMR (all P < 0.05) (Table 2).

**Table 2:** Comparison of CMR findings among T2DM patients with/without FMR and normal controls

	Normal (n = 52)	T2DM	
		Without FMR (n = 98)	With FMR (n = 59)
LV function parameters			
LVEDV, ml	121.1 ± 27.1	125.6 (104.3, 158.4)	172.6 (127.1, 261.3)*†
LVESV, ml	42.7 (37.2, 49.6)	45.6 (35.1, 62.2)	93.2 (52.6, 176.7)*†
LVSV, ml	76.4 ± 18.5	77.4 ± 23.5	75.0 (62.6, 85.9)
LVEF, %	62.6 ± 5.8	61.9 (57.0, 66.8)	42.2 (30.3, 60.3)*†
LV mass, g	72.1 ± 19.1	91.2 (70.4,115.0)*	111.3 ± 40.9*†
PS, %			
Radial	38.4 ± 8.7	31.3 ± 9.8*	18.3 ± 9.7*†
Circumferential	− 19.9 ± 2.7	− 19.6 (− 22.2, − 16.9)	− 11.8 ± 5.1*†
Longitudinal	− 14.8 ± 3.3	− 13.3 ± 3.2*	− 9.3 ± 3.4*†
PSSR, 1/s			
Radial	2.1 ± 0.7	1.7 (1.4, 2.1)*	1.0 (0.6, 1.5)*†
Circumferential	− 1.0 (− 1.1, − 0.9)	− 1.0 (− 1.2, − 0.8)	− 0.7 ± 0.3*†
Longitudinal	− 0.8 (− 0.9, − 0.6)	− 0.8 (− 0.9, − 0.6)	− 0.5 (− 0.7, − 0.4)*†
PDSR, 1/s			
Radial	− 2.8 ± 0.9	− 1.9 (− 2.4, − 1.3)*	− 1.2 (− 1.7, − 0.6)*†
Circumferential	1.2 ± 0.3	1.1 ± 0.3*	0.7 ± 0.3*†
Longitudinal	0.9 (0.8, 1.2)	0.8 (0.6, 1.1)	0.6 ± 0.2*†

*P < 0.05, T2DM vs. Normal; †P < 0.05, T2DM patients with FMR vs. T2DM patients without FMR. *T2DM* type 2 diabetes mellitus, *FMR* functional mitral regurgitation, *LV* left ventricular, *EDV* end diastolic volume, *ESV* end systolic volume, *SV* stroke volume, *EF* ejection fraction, *PS* peak strain, *PSSR* peak systolic strain rate, *PDSR* peak diastolic strain rate

### Characteristics of LV strain in patients with different regurgitation degrees

**Fig. 1:**
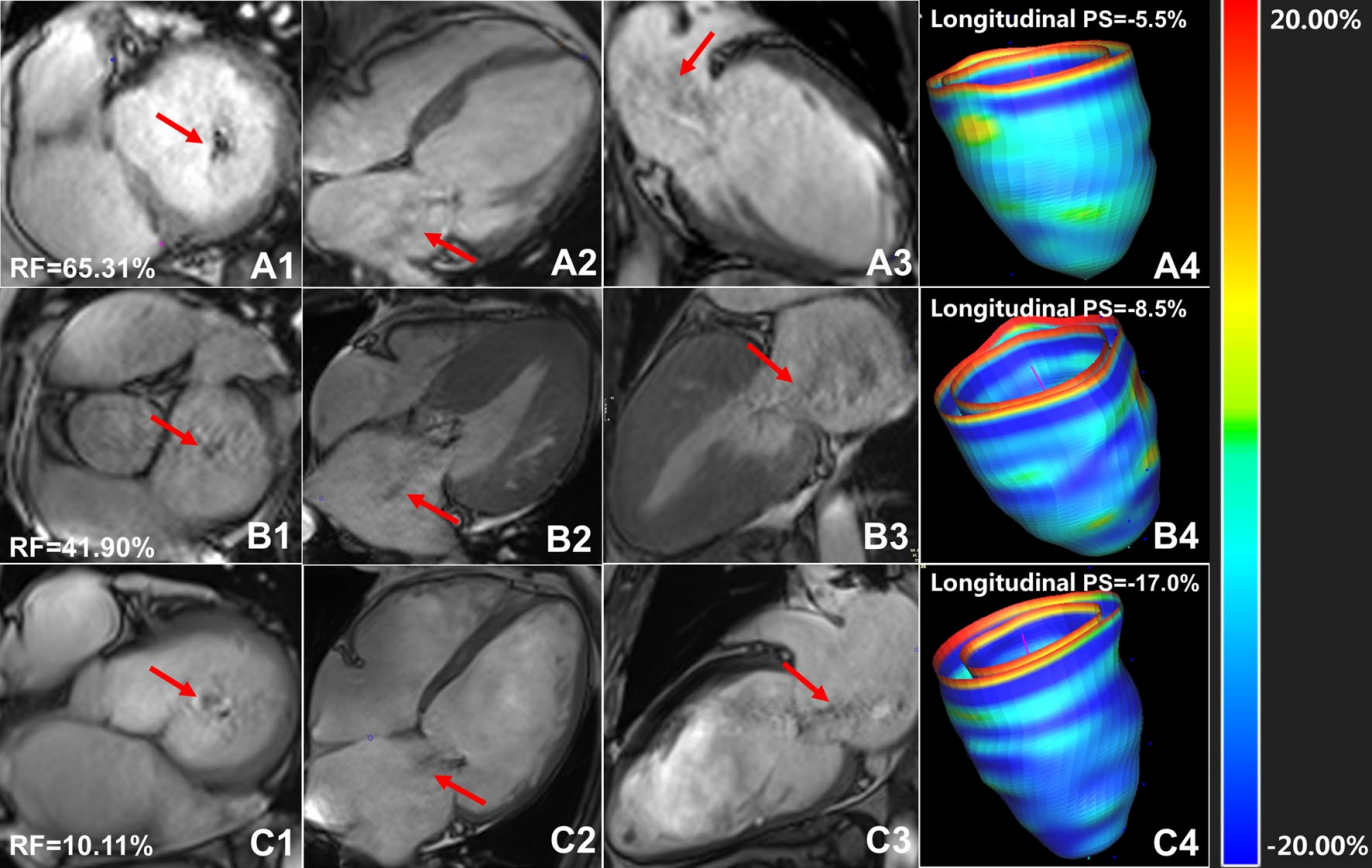
Cardiac cine images and three-dimensional pseudo-colour images of LV longitudinal strain in T2DM patients with mild, moderate and severe regurgitation. A1-3, T2DM patient with severe mitral regurgitation, female, 44 years old, left ventricular short axis (A1), four-chamber (A2), two-chamber (A3) cine sequence images showed large black regurgitation signal from left ventricle into left atrium (red arrow) reached the posterior wall of left atrium, RF = 65.31%; B1–3, T2DM with moderate mitral regurgitation, female, 58 years old, left ventricular short axis (B1), four-chamber (B2), two-chamber (B3) cine sequence images showed moderate mitral regurgitation (red arrow), RF = 41.90%; C1–3, T2DM patient with mild mitral regurgitation, male, 61 years old, left ventricular short axis (C1), four-chamber (C2), two-chamber (C3) cine sequence images showed a small regurgitation signal (red arrow), RF = 10.11%. A4, B4 and C4 were three-dimensional pseudo color maps of left ventricular longitudinal strain. T2DM, type 2 diabetes mellitus; RF, regurgitation fraction; PS, peak strain

Among T2DM patients with FMR, 21 patients (35.6%) had mild FMR, 19 patients (32.2%) had moderate FMR and 19 patients (32.2%) had severe FMR. The comparison of PS, PSSR and PDSR among the groups is shown in Table 3. Figure 1 shows cardiac cine images and three-dimensional pseudo-colour images of LV longitudinal strain in T2DM patients with mild, moderate and severe regurgitation.

**Table 3:** Comparison of LV strain among T2DM patients with mild/moderate/severe regurgitation and normal controls

	Normal (n = 52)	T2DM with mild FMR (n = 21)	T2DM with moderate FMR (n = 19)	T2DM with severe FMR (n = 19)
LVEF, %	62.6 ± 5.8	47.4 ± 12.0*	49.6 (28.6, 62.8)*	40.2 ± 19.5*
PS, %				
Radial	38.4 ± 8.7	22.8 ± 8.6*	16.1 ± 17.4*†	15.6 ± 10.3*†
Circumferential	− 19.9 ± 2.7	14.4 ± 4.2*	11.0 ± 5.1*†	8.9 (5.2, 13.8) *†
Longitudinal	− 14.8 ± 3.3	11.1 ± 3.2*	8.8 ± 3.4*†	7.2 (5.6, 10.9) *†
PSSR, 1/s				
Radial	2.1 ± 0.7	1.3 ± 0.6*	1.0 ± 0.5*	1.1 (0.5, 1.1) *†
Circumferential	− 1.0 (− 1.1, − 0.9)	− 0.8 ± 0.3	− 0.7 ± 0.3*	− 0.6 ± 0.3*
Longitudinal	− 0.8 (− 0.9, − 0.6)	− 0.7 (− 0.8, − 0.6)	− 0.5 ± 0.2*	− 0.4 (− 0.5, − 0.3) *†
PDSR, 1/s				
Radial	− 2.8 ± 0.9	− 1.5 ± 0.6*	− 1.0 ± 0.6*	− 0.9 ± 0.5*†
Circumferential	1.2 ± 0.3	0.9 ± 0.3*	0.7 ± 0.3*†	0.6 ± 0.3*†
Longitudinal	0.9 (0.8, 1.2)	0.6 (0.5, 1.0) *	0.6 ± 0.2*	0.5 ± 0.2*†

*P < 0.05, T2DM with FMR vs. normal; †P < 0.05, T2DM with severe/moderate FMR vs. T2DM with mild FMR

*T2DM* type 2 diabetes mellitus,*FMR* functional mitral regurgitation, *PS* peak strain, *PSSR* peak systolic strain rate, *PDSR* peak diastolic strain rate

In the mild group, the PS (radial, circumferential and longitudinal), PSSR (radial) and PDSR (radial, circumferential and longitudinal) were lower than those in the normal group (all P < 0.05). In the moderate group, the PS, PSSR and PDSR in all three directions were further decreased compared to those in the normal group (all P < 0.05). Compared with the mild, the PS (radial, circumferential and longitudinal) and PDSR (circumferential) were significantly decreased in the moderate (all P < 0.05). In the severe FMR group, the PS, PSSR and PDSR markedly decreased in all three directions compared with the normal (all P < 0.05). Compared with the mild, the PS (radial, circumferential and longitudinal), PSSR (radial and longitudinal) and PDSR (radial, circumferential and longitudinal) were all markedly decreased (all P < 0.05).

### Influencing factors of LV global PS in T2DM patients with FMR

**Fig. 2:**
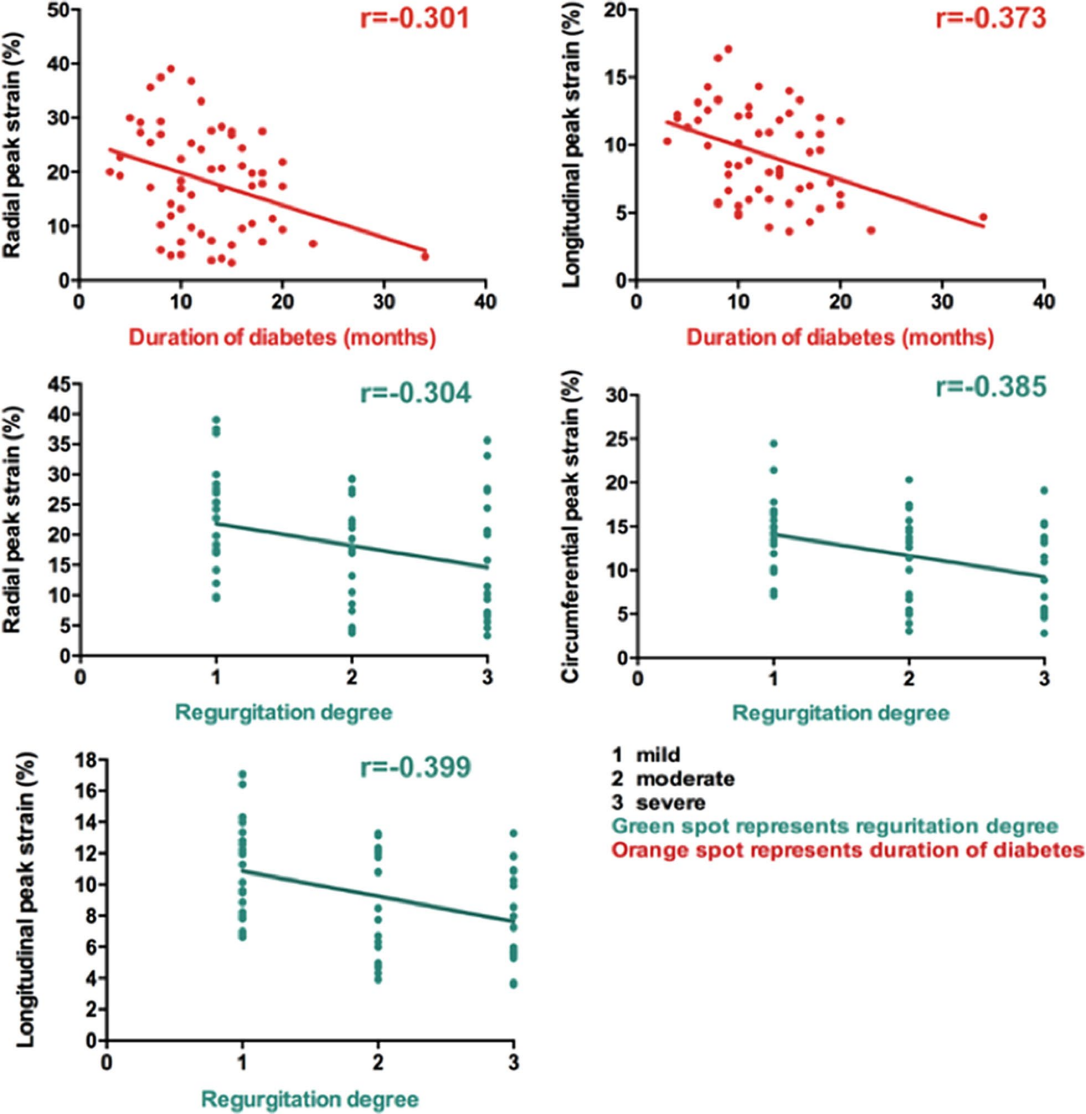
Correlation between the regurgitation degree of/months with diabetes and LV global PS. The absolute value of PS was used in the circumferential and longitudinal direction analysis to avoid the influence of directional sign. T2DM, type 2 diabetes mellitus; r, correlation coefficient

There was a negative correlation between the regurgitation degree and LV global radial PS (R = − 0.304, P = 0.019), circumferential PS (R = − 0.385, P = 0.003) and longitudinal PS (R = − 0.399, P = 0.002) in T2DM with FMR. In addition, a negative correlation was found between the months with diabetes and LV global radial PS (R = − 0.301, P = 0.021) and longitudinal PS (R = − 0.373, P = 0.004) but no correlation with circumferential PS (R = − 0.235, P = 0.073) in T2DM with FMR (Table 4). Figure 2 showed a scatter plot illustrating the correlation between the regurgitation degree of/months with diabetes and LV global PS.

**Table 4:** Univariable and multivariable analysis between the magnitude of LV peak strain and clinical indices in T2DM with FMR patients

	Radial PS				Circumferential PS				Longitudinal PS			
	Univariate	Multivariate			Univariate	Multivariate			Univariate	Multivariate		
	R	β (R^2^ = 0.185)	P	95% CI	R	β (R^2^ = 0.211)	P	95% CI	R	β (R^2^ = 0.271)	**P**	**95% CI**
Regurgitation degree	− 0.304a	− 0.272	0.029	− 6.043 to − 0.337	− 0.385a	− 0.412	0.001	− 3.975 to − 1.067	− 0.399a	− 0.347	0.004	− 2.393 to − 0.484
Months with diabetes	− 0.301a	− 0.299	0.017	− 0.975 to − 0.100	− 0.235	N/A	N/A	N/A	− 0.373a	− 0.347	0.004	− 0.366 to − 0.074
Fasting plasma glucose	0.091a	0.130	0.307	N/A	0.145	N/A	N/A	N/A	0.145	N/A	N/A	N/A
HbA1c	− 0.124	N/A	N/A	N/A	− 0.125	N/A	N/A	N/A	− 0.125	N/A	N/A	N/A
TC	0.126	N/A	N/A	N/A	0.089a	0.203	0.086	N/A	0.089a	0.094	0.437	N/A
TG	0.201	N/A	N/A	N/A	0.205	N/A	N/A	N/A	0.205	N/A	N/A	N/A
HDL	0.164	N/A	N/A	N/A	0.182	N/A	N/A	N/A	0.182	N/A	N/A	N/A
LDL	− 0.051a	0.143	0.266	N/A	− 0.097a	0.152	0.216	N/A	− 0.097a	0.959	0.628	N/A

a, *P* < 0.1 were included in the stepwise multiple linear regression model. Adjusted by gender, sex, age, BMI, systolic blood pressure, and rest heart rate

*PS* peak strain, *HbA1c* glycated haemoglobin, *TC* total cholesterol, *TG* triglycerides, *HDL* high-density lipoprotein, *LDL* low-density lipoprotein, *CI* confidence intervals

After adjusting for age, sex, BMI, systolic blood pressure and resting heart rate, multiple linear regression analysis further showed that the regurgitation degree was an independent factor of LV global radial (β = − 0.272), circumferential (β = − 0.412) and longitudinal (β = − 0.347) PS; the months with diabetes was an independent factor of LV global radial (β = − 0.299) and longitudinal (β = − 0.347) PS in T2DM with FMR (Table 4).

### Reproducibility of LV PS

Intra group correlation coefficient analysis showed that the CMR tissue-tracking technique in measuring LV radial PS (ICC: within observer, 0.937–0.979; between observers, 0.832–0.940), circumferential PS (ICC: within observer, 0.922–0.975; between observers, 0.799–0.927), longitudinal PS (ICC: within observer, 0.967–0.989; between observers, 0.907–0.968), radial PSSR (ICC: within observer, 0.847–0.950; between observers, 0.648–0.863), circumferential PSSR (ICC: within observer, 0.936–0.979; between observers, 0.830–0.940), longitudinal PSSR (ICC: within observer, 0.982–0.994; between observers, 0.949–0.983), radial PDSR (ICC: intra-observer, 0.859–0.954); inter-observer, 0.670–0.873), circumferential PDSR (ICC: intra-observer, 0.856–0.953); inter-observer, 0.664–0.870), longitudinal PDSR (ICC: intra-observer, 0.950–0.984); inter-observer, 0.865–0.953) had good intra- and inter-observer consistency.

## Discussion

The present study demonstrated that: (1) T2DM patients had decreased radial and longitudinal PS even without FMR; (2) when T2DM patients had FMR, the decrease in LV strain parameters was more severe in the radial, circumferential and longitudinal directions, and there was a significant decrease in LVEF; (3) along with the aggravation of regurgitation, the LV strain of T2DM patients with FMR decreased progressively, while diastolic dysfunction was mainly observed in mild and moderate group with systolic function being further damaged in the severe FMR group; and (4) the regurgitation degree was independently correlated with LV radial, circumferential and longitudinal PS, and the months with diabetes was independently correlated with LV radial and longitudinal PS in T2DM with FMR.

Fonseca et al. conducted a study using CMR strain and first reported the impairment of longitudinal and circumferential PS and PSSR in T2DM patients with normal LVEF [16]. Similarly, Vukomanovic et al. demonstrated that the longitudinal and circumferential strains of the LV in diabetic patients decrease significantly, affecting all layers of the LV [17]. In a large-scale meta-analysis, Kalam et al. proved that LV longitudinal strain is independently related to all-cause mortality and composite cardiovascular endpoint in patients with various heart diseases, and myocardial longitudinal strain is more reliable in predicting cardiovascular events than LVEF [10]. Consistent with previous studies, we found that T2DM without FMR had increased myocardial mass and decreased PS, PSSR and PDSR in the radial and longitudinal directions but similar LVEDV, LVESV, LVSV, and LVEF than normal controls. This may be explained that the myocardial changes of T2DM patients without FMR are mainly manifested as the pathological process of simple diabetic cardiomyopathy. Sustained hyperglycaemia can cause oxidative stress, renin angiotensin system activation, vascular endothelial dysfunction, cytokine activation and cardiac autonomic nervous dysfunction, which may lead to myocardial extracellular matrix deposition, increased oxygen consumption of myocardial cells, myocardial hypertrophy, myocardial interstitial fibrosis and decreased myocardial compliance, ultimately resulting in cardiac dysfunction [18, 19]. These results indicate that the variations in strain occur earlier and are more noticeable than LVEF, affirming that early detection of PS changes is important in constructive interventions and delaying the deterioration of the myocardium in T2DM patients.

In the present study, the T2DM with FMR had markedly decreased PS, PSSR and PDSR compared to both normal individuals and T2DM without FMR. The LVEDV, LVESV and LV mass were significantly increased, while the LVSV and LVEF were decreased in T2DM with FMR. A previous study on nondiabetic mitral regurgitation has demonstrated that LV dysfunction is a powerful predictor correlated with mitral regurgitation. Adjusting LV function may reduce the effect of mitral regurgitation on prognosis [16]. These results confirmed that FMR may aggravate myocardial stiffness, increase LV volume, decrease left ventricular compliance and induce LV dysfunction in T2DM patients. The aggravation of FMR on LV stiffness may be explained by the fact that FMR produces a pressure gradient between the left atrium and LV, resulting in LV volume overload, which can be compensated for a long period of time in the early stage to maintain cardiac output, but a sustained high-volume load eventually leads to myocardial dysfunction. The increase in stiffness is irreversible even if the regurgitation is corrected [3, 4, 20, 21].

By comparing T2DM with different regurgitation degree, we found that LV global PS showed progressive decrease from normal, the mild to the moderate and the severe FMR group, and the degree of myocardial strain damage was similar in moderate and severe mitral regurgitation group. In addition, multivariate analysis showed that the regurgitation degree was an independent risk factor for LV radial (β = − 0.272), circumferential (β = − 0.412) and longitudinal (β = − 0.347) PS. It was demonstrated that PS may decrease with the increase of regurgitation degree and the myocardial stiffness in patients with moderate and severe regurgitation increased significantly compared with the mild.

In addition, PSSR in mild group did not decline significantly, gradually began to decline in moderate group, and decreased further in the severe FMR group, while PDSR appeared in mild patients, decreased further in moderate patients and decreased markedly in severe patients. It may be explained that in T2DM patients with mild regurgitation, LV compensatory hypertrophy maintains systolic function in normal range, but the LV hypertrophy and stiffness leads to a decrease of diastolic function. With the development of regurgitation, the LV systolic function deteriorated significantly [21–23].

There are several limitations in this study. Firstly, this is a single centre study, but the sample size is enough to prove the additive effect of FMR on LV impairment in patients with T2DM. Secondly, a larger cohort or multicentre study with long-term follow-up data is required in the future. Thirdly, the present study did not carry out animal experiment, studies focus on the relevant pathological mechanism will be conducted in the future. Finally, subclinical coronary ischemic disease may not be excluded because the stress test was not performed. However, the clinical coronary artery disease was considered to be unlikely according to the evaluation of patients by clinical history, laboratory results, echocardiography and electrocardiography.

## Conclusions

FMR may aggravate the deterioration of LV stiffness and cause decrease of LV strain and cardiac dysfunction in T2DM patients. The regurgitation degree and months with diabetes were independently correlated with LV PS in T2DM with FMR. The evaluation of LV strain may facilitate clinicians to monitor the progression of myocardial stiffness and make additional strategies to delay the LV dysfunction in T2DM with FMR.

## Supplementary Information

**Additional file 1:** **Fig. S1. **Analysis of left ventricular volume and function by cardiovascular magnetic resonance cine images. The left ventricular endocardium (red) and epicardium (green) were outlined and the papillary muscle was excluded on the left ventricular short axis images of end diastolic (A) and end systolic (B), two-chamber long axis (C) and four-chamber long axis (D) images of end diastolic. The blue T-line defines the mitral plane and apex. Figure E shows the 3D volume model of left ventricle automatically established.

**Additional file 2:** **Fig. S2**. Qualitative diagnosis of mitral regurgitation and quantitative calculation of regurgitation fraction. On the short axis (A), four-chamber long axis (B) and two-chamber long axis (C) of cardiac MR cine images, the white arrow showed the black regurgitation signal at the mitral valve orifice. The right ventricular endocardium and left ventricular endocardium were outlined at end systolic (D) and end diastolic (E) images and the pink curve showed the exclusion of papillary muscle. Using the following formula calculate the regurgitation fraction. LVEDV, left ventricular end diastolic volume; LVESV, left ventricular end systolic volume; RVEDV, right ventricular end diastolic volume; RVESV, right ventricular end systolic volume; LVSV, left ventricular stroke volume; RVSV, right ventricular stroke volume; RF, regurgitation fraction.

## Data Availability

The datasets used and analyzed during the current study are available from the corresponding author on reasonable request.

## Abbreviations

FMR: Functional mitral regurgitation
T2DM: Type 2 diabetes diabetes mellitus
LV: Left ventricle
CMR: Cardiac magnetic resonance
LVEF: Left ventricle ejection fraction
PS: Peak strain
HbA1c: Glycated haemoglobin
HDL: High density lipoprotein
LDL: Low density lipoprotein
BMI: Body mass index
RV: Right ventricle
EDV: End-diastolic volume
ESV: End systolic volume
SV: Stroke volume
LVEF: Left ventricle ejection fraction
RF: Regurgitation fraction
PSSR: Peak systolic strain rate
PDSR: Peak diastolic strain rate
ICC: Intraclass correlation coefficient
