# Supplementary figures and images for: Aggravation of functional mitral regurgitation on left ventricle stiffness in type 2 diabetes mellitus patients evaluated by CMR tissue tracking
Source: Cardiovasc Diabetol. 2021 Jul 31;20:158. doi: 10.1186/s12933-021-01354-y (PMC8325822; doi:10.1186/s12933-021-01354-y)

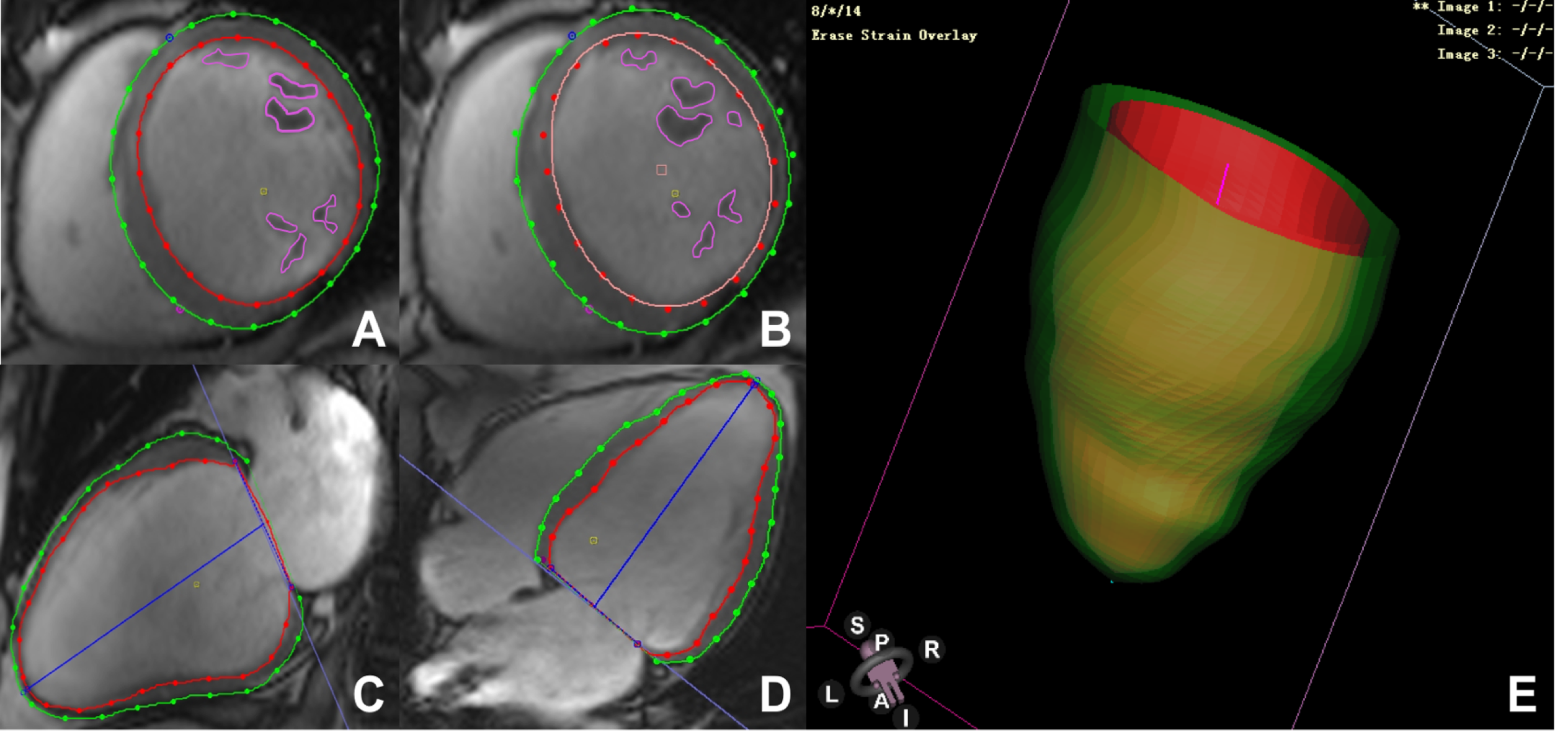

Supplement: Supplementary file 1 — Additional file 1: Fig. S1. Analysis of left ventricular volume and function by cardiovascular magnetic resonance cine images. The left ventricular endocardium (red) and epicardium (green) were outlined and the papillary muscle was excluded on the left ventricular short axis images of end diastolic (A) and end systolic (B), two-chamber long axis (C) and four-chamber long axis (D) images of end diastolic. The blue T-line defines the mitral plane and apex. Figure E shows the 3D volume model of left ventricle automatically established. [file 12933_2021_1354_MOESM1_ESM.tif]

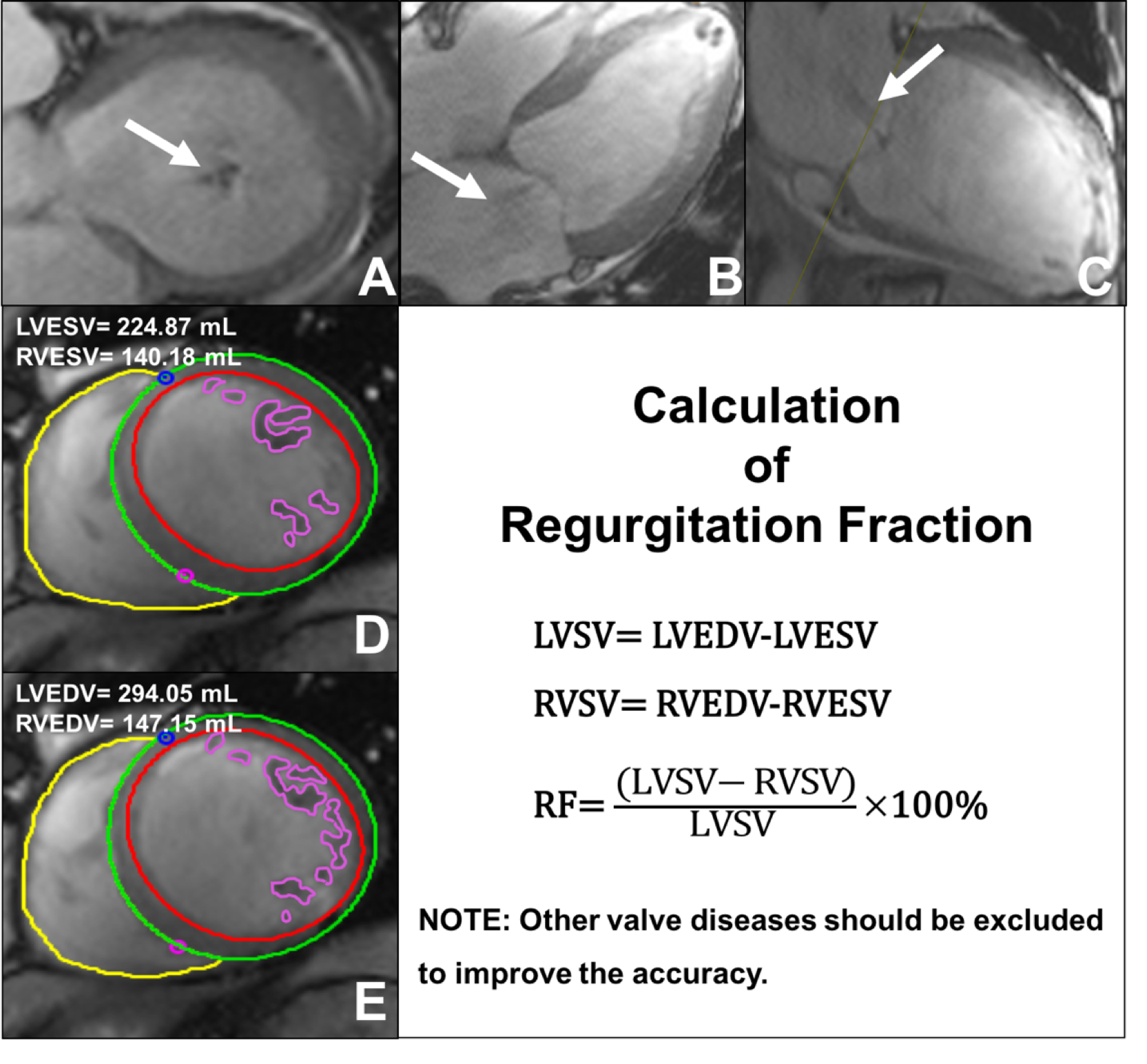

Supplement: Supplementary file 2 — Additional file 2: Fig. S2. Qualitative diagnosis of mitral regurgitation and quantitative calculation of regurgitation fraction. On the short axis (A), four-chamber long axis (B) and two-chamber long axis (C) of cardiac MR cine images, the white arrow showed the black regurgitation signal at the mitral valve orifice. The right ventricular endocardium and left ventricular endocardium were outlined at end systolic (D) and end diastolic (E) images and the pink curve showed the exclusion of papillary muscle. Using the following formula calculate the regurgitation fraction. LVEDV, left ventricular end diastolic volume; LVESV, left ventricular end systolic volume; RVEDV, right ventricular end diastolic volume; RVESV, right ventricular end systolic volume; LVSV, left ventricular stroke volume; RVSV, right ventricular stroke volume; RF, regurgitation fraction. [file 12933_2021_1354_MOESM2_ESM.tif]
